# New Oligoneuriidae (Insecta, Ephemeroptera) from Iran

**DOI:** 10.3897/zookeys.872.36098

**Published:** 2019-08-26

**Authors:** Pavel Sroka, Jindřiška Bojková, Roman J. Godunko, Tomáš Soldán, Javid Imanpour Namin, Farshad Nejat, Ashgar Abdoli, Arnold H. Staniczek

**Affiliations:** 1 Biology Centre of the Czech Academy of Sciences, Institute of Entomology, Branišovská 31, 37005 České Budějovice, Czech Republic Biology Centre of the Czech Academy of Sciences České Budějovice Czech Republic; 2 Department of Botany and Zoology, Masaryk University, Kotlářská 2, 61137 Brno, Czech Republic Masaryk University Brno Czech Republic; 3 Department of Invertebrate Zoology and Hydrobiology, University of Łódź, Banacha 12/16, 90237 Łódź, Poland University of Łódź Łódź Poland; 4 Department of Fishery, Faculty of Natural Resources, University of Guilan, POB 1144, Sowmehsara-Rasht, Iran University of Guilan Sowmehsara-Rasht Iran; 5 Department of Biodiversity and Ecosystem Management, Environmental Sciences Research Institute, Shahid Beheshti University, Daneshjou Boulevard, 1983969411 Tehran, Iran Shahid Beheshti University Tehran Iran; 6 Department of Entomology, State Museum of Natural History Stuttgart, Rosenstein 1, 70191 Stuttgart, Germany State Museum of Natural History Stuttgart Stuttgart Germany

**Keywords:** Barcoding, mayflies, Middle East, *
Oligoneuriella
*, *
Oligoneuriopsis
*, taxonomy, new species

## Abstract

Two new species of the mayfly family Oligoneuriidae are described based on larval specimens recently collected in Iran. The first new species, *Oligoneuriella tuberculata* Godunko & Staniczek, **sp. nov.**, can be distinguished from all its congeners by the presence of pronounced protuberances posteromedially on abdominal terga, highly reduced paracercus, large lamella of gill I, and setation on hind margin of middle and hind femora confined to their basal halves. The second species, *Oligoneuriopsis villosus* Bojková, Godunko, & Staniczek, **sp. nov.**, remarkably belongs to a mostly Afrotropical genus. The new species clearly differs from all its congeners in the shape of setae on the surface of gills and terga, pattern of body colouration, and the shape of posterolateral projections of abdominal segments. Except for the species description, the generic diagnosis of *Oligoneuriopsis* Crass, 1947 is briefly discussed. COI barcode sequences of both new species are provided and molecular species delimitation is tested using distance-based and likelihood-based approaches, with both new species unambiguously recognised as separate lineages. The analysis of COI also corroborates the respective affinities of both new species, estimated based on morphology. The two new species of Oligoneuriidae described herein highlight the importance of the Middle East as a centre of diversity of this mayfly family within the Palaearctic.

## Introduction

The mayfly fauna of Iran is still largely unexplored, although considerable progress has been achieved recently. Bojková et al. (2018) summarised the investigations on Iranian mayflies published to date to initiate a more systematic research on the topic. During an extensive field trip in 2017, new material of the family Oligoneuriidae was collected throughout Iran. Within this material, two new species were recognised, attributable to the genera *Oligoneuriella* Ulmer, 1924 and *Oligoneuriopsis* Crass, 1947 (generic concepts according to Bauernfeind and Soldán 2012).

*Oligoneuriella* occurs in the Palaearctic and comprises 14 species (Sroka et al. 2015). The Middle East hosts a relatively high diversity of *Oligoneuriella*, with six species recorded exclusively in this area (*O. bicaudata* Al-Zubaidi, Braasch & Al-Kayatt, 1987, *O. orontensis* Koch, 1980, *O. tskhomelidzei* Sowa & Zosidze, 1973, *O. magna* Bojková & Soldán, 2015, *O. paulopilosa* Sroka, 2015, *O. pectinata* Bojková & Soldán, 2015) and another two species occurring in large parts of the Palearctic, namely *O. rhenana* (Imhoff, 1852) and *O. pallida* (Hagen, 1855).

*Oligoneuriopsis*, the second genus, which we found in Iran, is probably closely related to *Oligoneuriella* (Edmunds 1961; Massariol 2017). The former genus includes six species, mostly distributed in the Afrotropics (Bauernfeind and Soldán 2012), with the only exception of *Oligoneuriopsis skhounate* Dakki & Giudicelli, 1980, which is known from the Palaearctic (North Africa and the Iberian Peninsula). It has to be mentioned that the delimitation of *Oligoneuriopsis* is problematic and its separation from *Oligoneuriella* is questionable (see Bauernfeind and Soldán 2012).

As a part of our study, we include new COI barcode sequences of *O. tskhomelidzei* and the two new species. We analyse these sequences to test the delimitation of the newly proposed species and compare them with all *Oligoneuriopsis* and *Oligoneuriella* species, for which COI sequences are available, namely *O. bicaudata*, *O. pallida*, *O. rhenana*, *O. skhounate*, and two unidentified species from Iraq and China.

## Materials and methods

### Collecting and morphological study

Material used for this study was collected by J. Bojková, R.J. Godunko, J. Imanpour Namin, F. Nejat, M. Pallmann, T. Soldán, and A.H. Staniczek during an investigation of different freshwater habitats in Iran. Samples were obtained by kick sampling and specimens were preserved in 96% EtOH. Environmental variables (pH, conductivity, salinity and temperature) were measured using a HACH sensION 5 portable waterproof conductivity meter and HACH Pocket Pro+ Multi 2. Some specimens were dissected and mounted on slides with HydroMatrix (MicroTech Lab, Graz, Austria) to allow detailed microscopic observations. Drawings were made using a stereomicroscope Olympus SZX7 and a microscope Olympus BX41, both equipped with a drawing attachment. Serial habitus photographs were made with a Leica DMC5400 digital camera on a Leica Z16 APO Macroscope using Leica Application Suite Version 3.1.8 and Helicon Focus Pro to obtain stacked photographs with extended depth of field.

For scanning electron microscopy (SEM), eggs were dissected from female last instar larvae and also mouthparts, legs, and gills were dissected. All parts were subsequently dehydrated through a stepwise immersion in ethanol, dried by critical point drying (Leica EM CPD300), and mounted on SEM stubs. The mounted material was coated with a 5 nm Au/Pd layer (Leica EM ACE200) and subsequently examined and photographed with a Zeiss EVO LS 15 scanning electron microscope. All photographs were subsequently sharpened and adjusted in contrast and tonality in Adobe PhotoshopTM CS6.

Material is deposited in the collections of the Biology Centre CAS, Institute of Entomology, České Budějovice, Czech Republic (**IECA**), State Museum of Natural History, Stuttgart, Germany (**SMNS**), State Museum of Natural History, National Academy of Sciences of Ukraine, Lviv, Ukraine (**SNHM**), and the Natural History Museum and Genetic Resources, Department of Environment, Teheran, Iran (**MMTT_DOE**).

### DNA extraction, PCR amplification, and sequencing

For DNA extraction, one severed leg of each specimen was processed with Qiagen DNeasy Blood & Tissue Kit following the manufacturer’s instructions for tissue samples. The resulting DNA-elute was used in a PCR reaction with the primer pair of LCO1490 and HCO2198 for COI targeting, utilising a Thermo Scientific™ Phire Tissue Direct PCR Master Mix Kit for samples of *O. tskhomelidzei* and a Qiagen Multiplex PCR Kit for the two new species. The PCR amplification was performed as follows: Initial heat activation at 95 °C for 15 min and 35 cycles of denaturation at 94 °C for 0:30 min. For Thermo Scientific protocol, annealing was performed at 50 °C for 1:30 min and elongation at 68 °C for 0:45 min, for Qiagen protocol, annealing was performed at 56 °C for 1:30 min and elongation at 72 °C for 1:30 min. Both protocols followed a final elongation step at 72 °C for 10 min. The PCR products were enzymatically purified using ExoI/FAP. The purified product was sequenced via the EZ-seq single direct service by Macrogen (Amsterdam, Netherlands). Resulting chromatograms were assembled and final sequences were error checked using Geneious suite version 10.2.3. COI sequences were deposited at Barcode of Life Data Systems (http://www.boldsystems.org) under accession numbers specified in Table 1.

**Table 1.** List of specimens used for the COI analysis.

**Species**	**Location**	**Voucher specimen collection code**	**Bold Process ID**	**GenBank accessionnumber**	**Source**
*O. rhenana*	Germany	–	GBEPT372-14	KY261779	GenBank
	Italy	–	GBA22942-15	LN734762	GenBank
	Italy	–	GBA22943-15	LN734763	GenBank
	France	–	GBMIN65367-17	MF458765	GenBank
	Germany	–	GBEPT1975-14	KY261297	GenBank
	France	–	GBMIN65371-17	MF458760	GenBank
	Germany	–	GBEPT344-14	KY262278	GenBank
	France	–	GBMIN65370-17	MF458763	GenBank
	Germany	–	GBEPT1221-14	KY261341	GenBank
	Germany	–	FBAQU839-10	KY262106	GenBank
	Germany	–	FBAQU1261-12	KY262260	GenBank
*O. pallida*	Hungary	–	–	–	Massariol (2017)
	Hungary	–	GBMIN65365-17	KU609047	GenBank
*O. tskhomelidzei*	Iran	SMNS_EPH_7724_V_1	EPHIR014-19	–	newly sequenced
	Iran	SMNS_EPH_7596_V_4	EPHIR012-19	–	newly sequenced
	Iran	SMNS_EPH_7596_V_6	EPHIR013-19	–	newly sequenced
*O. bicaudata*	Iraq	–	BMIKU058-09	–	Bold Systems
	Iraq	–	BMIKU056-09	–	Bold Systems
	Iraq	–	BMIKU054-09	–	Bold Systems
*O. tuberculata* sp. nov.	Iran	SMNS_EPH_7574_V_1	EPHIR003-19	–	newly sequenced
	Iran	SMNS_EPH_7574_V_2	EPHIR004-19	–	newly sequenced
	Iran	SMNS_EPH_7574_V_4	EPHIR005-19	–	newly sequenced
	Iran	SMNS_EPH_7574_V_5	EPHIR006-19	–	newly sequenced
	Iran	SMNS_EPH_7574_V_6	EPHIR002-18	–	newly sequenced
*Oligoneuriella* sp. 1	Iraq	–	BMIKU059-09	–	Bold Systems
	Iraq	–	BMIKU040-09	–	Bold Systems
	Iraq	–	BMIKU037-09	–	Bold Systems
	Iraq	–	BMIKU031-09	–	Bold Systems
*Oligoneuriella* sp. 2	China	–	XJDQD857-18	–	Bold Systems
	China	–	XJDQD856-08	–	Bold Systems
*O. skhounate*	Spain	–	–	–	Massariol (2017)
*O. villosus* sp. nov.	Iran	SMNS_EPH_7550_V_2	EPHIR008-19	–	newly sequenced
	Iran	SMNS_EPH_7555_V_3	EPHIR011-19	–	newly sequenced
	Iran	SMNS_EPH_7555_V_2	EPHIR010-19	–	newly sequenced
	Iran	SMNS_EPH_7550_V_4	EPHIR009-19	–	newly sequenced
	Iran	SMNS_EPH_7550_V_1	EPHIR007-19	–	newly sequenced

### Analysis of sequences

We analysed COI to test the validity of the morphological species concept of both new species. COI of *O. tuberculata* sp. nov., *O. villosus* sp. nov., and *O. tskhomelidzei* were newly sequenced. Remaining sequences used in the analysed dataset were downloaded from GenBank and Bold Systems. Two sequences (one from *Oligoneuriopsis skhounate* and one from *Oligoneuriella pallida*) were obtained from Massariol (2017). Identical haplotypes were collapsed. Final alignment was constructed and edited using Geneious 11.0.4 (http://www.geneious.com) and contained fragments 359–655bp long (for details and accession numbers of individual sequences see Table 1).

To split a sequences alignment dataset into candidate species, the alignment was analysed using Automatic Barcode Gap Discovery (ABGD) (Puillandre et al. 2012) (http://wwwabi.snv.jussieu.fr/public/abgd/). This clustering method calculates the distance matrix and identifies the so-called barcode gap that would correspond to the threshold between intra- and interspecific genetic distances (Kekkonen et al. 2015). The settings were default, except for the X value (relative gap width), which was set to 1.0. The Jukes-Cantor (JC69) model was used, selected as best substitution model according to AICc in JmodelTest 2.1.10 (Darriba et al. 2012). The distances within and between recognised species were calculated in MEGA 7 (Kumar et al. 2016).

Species delimitation was also tested using single-loci coalescence based General Mixed Yule Coalescent model (GMYC) (Fujisawa and Barraclough 2013). The GMYC represents a model-based approach, aiming to discover the maximum likelihood solution for the threshold between the branching rates of speciation and coalescent processes on a tree (Kekkonen et al. 2015). The likelihood ratio test assesses if the mixed model fits the data significantly better than a null model that assumes a single coalescent process for the entire tree (Vuataz et al. 2011). Analyses were performed using the SPLITS package for R (http://r-forge.r-project.org/projects/splits).

An ultrametric COI gene tree was reconstructed under a relaxed molecular clock (uncorrelated lognormal distribution) using BEAST 2.4.8 (Bouckaert et al. 2014). An input file was generated in BEAUti 2. The best substitution model (from the models available in BEAST) was determined according to AICc as GTR using JmodelTest 2.1.10. A coalescent constant size tree prior was preferred, because the GMYC null model constitutes a single coalescent cluster (Vuataz et al. 2011, 2013). Other settings were default. MCMC chains were run for 50 million generations sampled every 5000 generations. Convergence and effective sample size were verified using Tracer 1.6. The first 10% of trees (1000) were discarded as burn-in. The maximum clade credibility tree was constructed from 9000 trees using TreeAnnotator 1.8.4 with default settings and visualised using FigTree 1.4.3.

## Results and discussion

### Oligoneuriella tuberculata

Taxon classificationAnimaliaEphemeropteraOligoneuriidae

Godunko & Staniczek sp. nov.

B12E2AEB8ACB5D72972A83E4DE337FE5

http://zoobank.org/6CE696BD-36EF-443B-87FC-DB92E0E135C1

**Figure 1.**
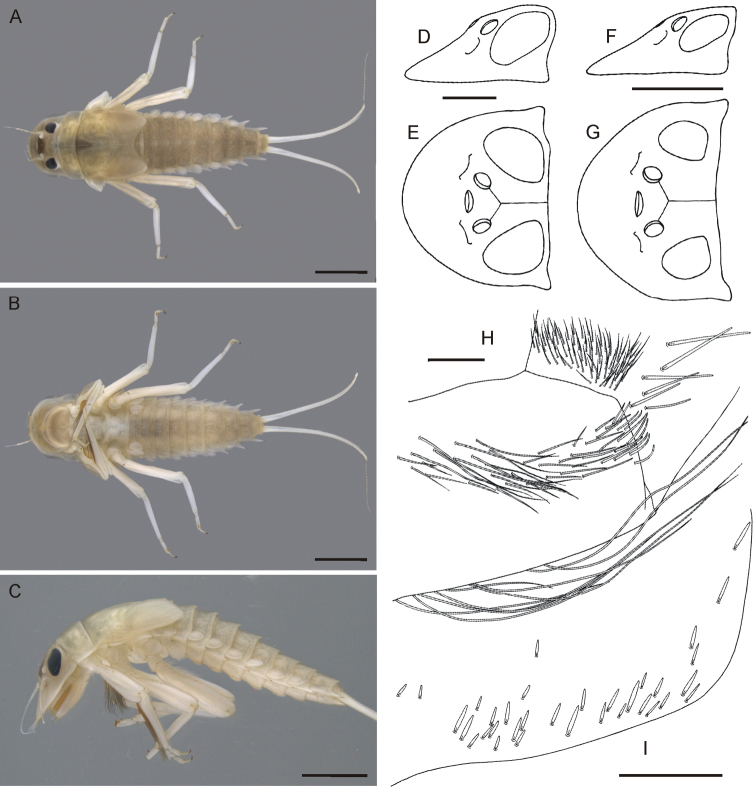
*Oligoneuriella tuberculata* sp. nov., larvae **A** habitus in dorsal view **B** habitus in ventral view **C** habitus in lateral view **D** head of male in lateral view (immature larva) **E** head of male in dorsal view (immature larva) **F** head of female in lateral view **G** head of female in dorsal view **H** detail of setae on distal part of labial palp segment I in dorsal view **I** detail of setae on proximal margin of paraglossae in ventral view. Scale bars: 2.0 mm (**A–C**); 0.5 mm (**D–G**); 0.1 mm (**H**); 0.2 mm (**I**). Same scale bar for **D** and **E**. Same scale bar for **F** and **G**.

**Figure 2.**
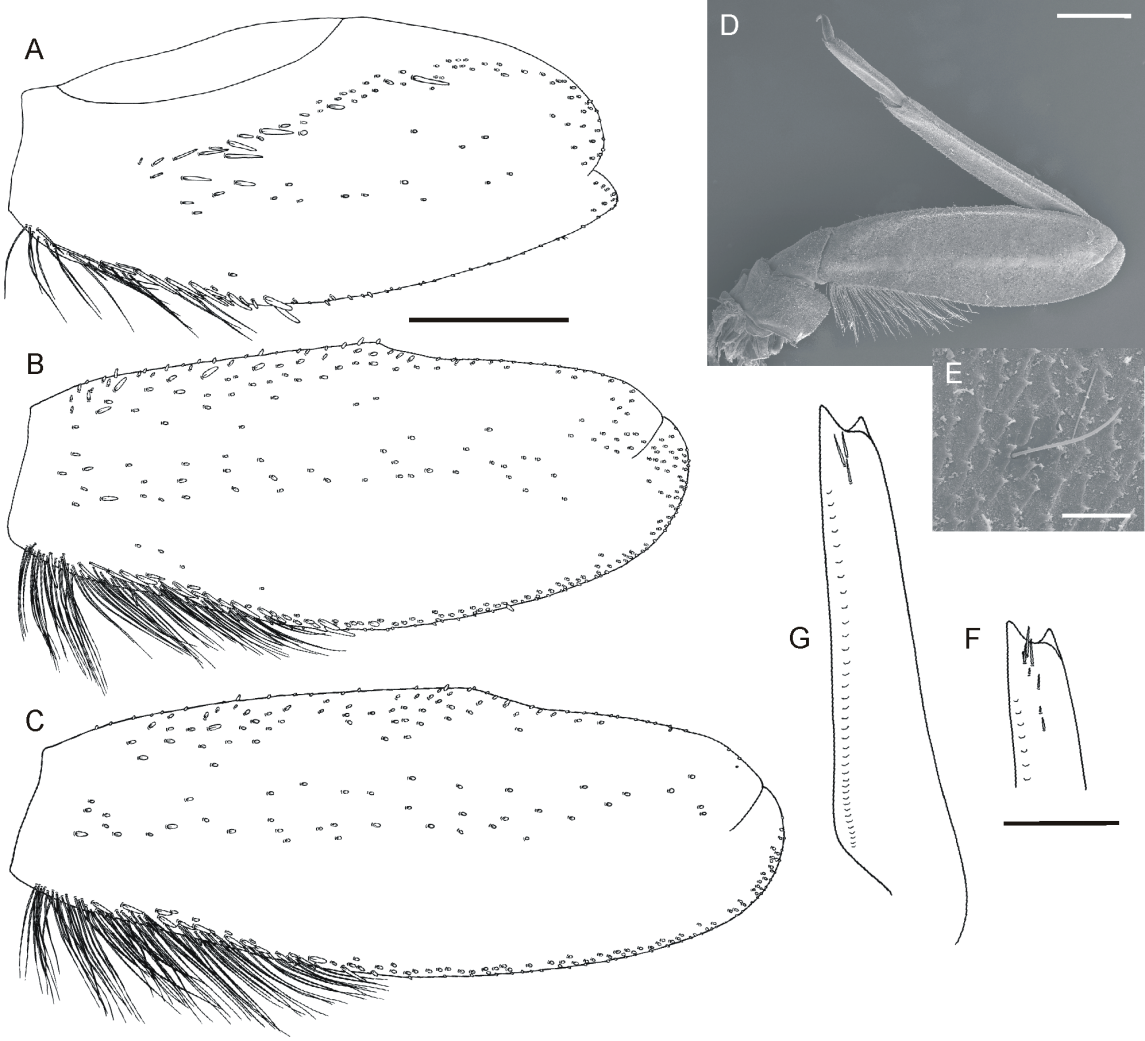
*Oligoneuriella tuberculata* sp. nov., larvae, legs **A** forefemur in dorsal view **B** middle femur in dorsal view **C** hind femur in dorsal view **D** middle leg under SEM in dorsal view **E** micro sculpture of leg cuticula under SEM in dorsal view **F** apical part of foretibia with subapical setae in ventral view **G** shape of foretibia with subapical setae in dorsal view. Scale bars: 0.5 mm (**A–D, F, G**); 0.02 mm (**E**). Same scale bar for **A–C**. Same scale bar for **F** and **G**.

**Figure 3.**
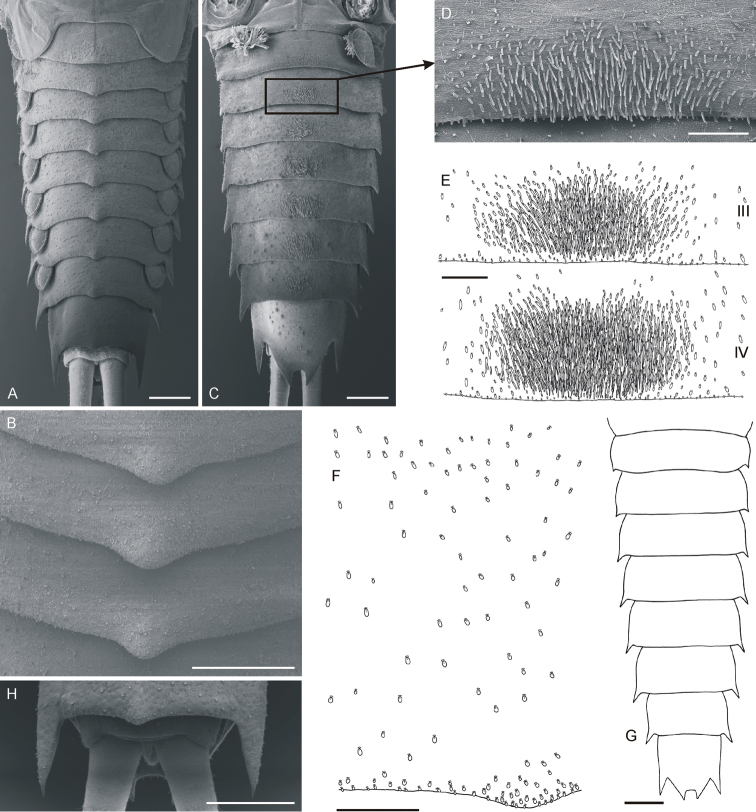
*Oligoneuriella tuberculata* sp. nov., larvae, abdomen **A** abdomen under SEM in dorsal view **B** detail of tergal protuberances under SEM**C** abdomen under SEM in ventral view **D** detail of setae on sternum III under SEM**E** detail of setae on sterna III and IV **F** detail of setae on surface of terga **G** outline of abdomen. **H** detail of abdomen tip under SEM with bases of cerci and vestigial paracercus. Scale bars: 0.5 mm (**A–C, H**); 0.2 mm (**D–F**); 1 mm (**G**).

**Figure 4.**
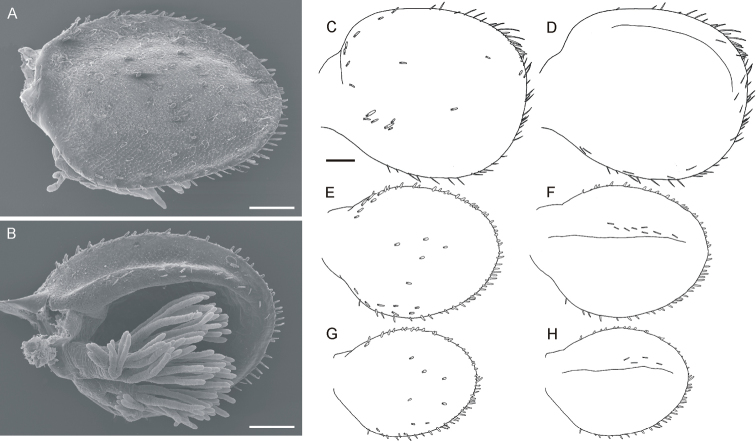
*Oligoneuriella tuberculata* sp. nov., larvae, gills **A** gill IV under SEM in dorsal view **B** gill IV under SEM in ventral view **C** gill plate I in dorsal view **D** gill plate I in ventral view **E** gill plate IV in dorsal view **F** gill plate IV in ventral view **G** gill plate VII in dorsal view **H** gill plate VII in ventral view. Scale bars: 0.1 mm (**A–H**). Same scale bar for **C–H**.

Figures 1 , 2 , 3 , 4 , Table 2

#### Etymology.

The name of the new species refers to the presence of protuberances posteromedially on terga, which is a character unknown for any other species of *Oligoneuriella*. A tuberculum is the Latin expression for protuberance.

#### Type material.

***Holotype***: Male larva, IRAN, Kohgiluyeh and Boyer-Ahmad Province, Kata, Marbor River, 31°10.71'N, 51°15.78'E, 1562 m a.s.l., 04.05.2017, leg. Staniczek A.H., Godunko R.J., Pallmann M. & Nejat, F. The holotype is deposited at SMNS under inventory number SMNS_EPH_7574_B_3.

***Paratypes***: 165 larvae, same locality as holotype (40 larvae deposited in SMNS under inventory numbers SMNS_EPH_7574_B_1 and SMNS_EPH_7574_B_2, including DNA voucher specimens with inventory numbers specified in Table 1, 20 larvae deposited in IECA, 100 larvae deposited in SNHM, and 5 larvae deposited in MMTT_DOE).

**Table 2.** Summary of main larval characters of *O. villosus* sp. nov. and *O. tuberculata* sp. nov.

	***O. villosus* sp. nov.**	***O. tuberculata* sp. nov.**
Body length (mm)	13–16	>9–12 (fully mature larvae not available)
Colour pattern	greyish dark brown with distinctive light (yellowish) ornamentation	yellowish-white, light brown to light dirty olivaceous, without marked ornamentation
Compound eyes of male exceeding / not exceeding head margin laterally	slightly exceeding	unknown (fully mature male larvae not available)
Basal setae on paraglossae	sparse, slightly elongated, not arranged in rows	sparse, slightly elongated, not arranged in rows
Setae distally on segment I of labial palps	approx. 15–25, short, hair-like	more than 30, short, hair-like
Hair-like setae proximally on posterior margin of middle- and hind femora	dense and long, forming fringe along all length of femur	dense and long, forming fringe, reaching 1/3–1/2 of femur length
Submedial row of spine-like setae dorsally on fore tibiae	irregular row of bristle-like setae in distal quarter	3–4 setae arranged in irregular row subapically
Presence of posterolateral projections on abdominal segments	II–IX	(II) III–IX
Shape of posterolateral projections of last abdominal segments	bent outwards, apices slightly inwards	nearly straight, slightly diverging from body axis
Posteromedial setae on sterna III-IV	some more than 20× longer than wide	up to 10–15× longer than wide
Size of first gill plate compared to remaining pairs	markedly smaller	significantly larger
Setae on inner distal margin of gill plates II–VII	long	slightly elongated
Setae on ventral surface of gill plates near inner distal margin	present	absent
Paracercus	fully developed	vestigial

#### Localities and biology.

The single known locality of *O. tuberculata* sp. nov. in Marbor River is situated in the southeastern part of the Zagros Mountains within the Khersaan River basin (Fig. 9), in close proximity to the Dena Protected Area. The majority of the drainage area is used as pasturage for a small amount of livestock, in nearer surroundings the land use is mainly for farming and rural, residential purposes.

Marbor River at the type locality is a small, premontane river, 15–22 m wide, with depths up to 1.5–1.7 m (Fig. 10A). The river bed is braided and formed of coarse and fine gravel, with sparse cobbles. At the type locality the river formed two approximately equal branches with fast, turbulent flow (0.4–0.9 m/s), and a wide alluvium. The larvae inhabited the gravel substratum in the fast flow of the streamline, but were also found in the littoral zone.

Water quality at Marbor River was good, with conductivity 264 µS.cm, salinity 0.1 ‰, and temperature 15 °C.

The emergence of the species can be expected approximately between July and August, as all larvae collected here in May were small and only half-grown with small wing pads. Together with *O. tuberculata* sp. nov., only three other mayfly taxa were collected: Baetis (Rhodobaetis) sp. (Baetidae), *Rhithrogena* sp. (Heptageniidae) and *Epeorus* sp. (Heptageniidae).

#### Diagnosis.

According to the combination of following diagnostic characters, *O. tuberculata* sp. nov. can be distinguished from all other representatives of the genus *Oligoneuriella* worldwide:

• Body pale, abdominal terga with inconspicuous, pale maculae laterally (Fig. 1A);

• eyes of male laterally not exceeding head margin (Fig. 1A, E);

• dorsal side of foretibia with 3–4 setae arranged into irregular row subapically (Fig. 2G);

• dense rows of long, hair-like setae on posterior margin of proximal 1/3–1/2 of middle- and hind femora (Fig. 2B, C);

• posterolateral processes of abdominal segments relatively narrow (Fig. 3G);

• terga II–IX with unpaired protuberance posteromedially (Fig. 3A, B);

• posteromedial flattened setae on sterna III–IV up to 10–15× longer than wide (Fig. 3D, E);

• first gill plate significantly larger than remaining gill plates, without ridge near outer margin dorsally (Fig. 4C, D);

• sparse setation on dorsal and ventral surfaces of gill plates II–VII (Fig. 4A, B, E–H);

• ventral surface of gill plates II–VII with setae sparsely scattered only along outer margin of ventral cavity (Fig. 4B, F, H);

• paracercus vestigial, approximately 5-segmented.

#### Description.

***Larva.*** Submature larvae: body length 9–12 mm (female), 8–9 mm (male), length of cerci approximately 0.70–0.85× body length, paracercus vestigial, 5-segmented.

*Colouration* (Fig. 1A–C). General colour pale, yellowish-white, light brown to light dirty olivaceous; dorsal side darker than ventral one; pronotum with 2–3 small, pale, diffuse spots centrally; pro- and mesosternum whitish-yellow; abdominal terga with inconspicuous, pale maculae laterally; sterna uniformly yellowish.

*Head*. Pale, yellowish-brown to dirty olive, slightly darker centrally; ocellar area brown. Antennae unicoloured, yellow. Head width/length 1 : 1.1–1.3 in submature larvae (Fig. 1D–G). Eyes black, relatively elongated, not exceeding contour of head in dorsal view in male submature larvae (Fig. 1E); distance between eyes 0.5× narrower than eye width in male and about 1.2× than eye width in female. Foremargin of head pale yellow, bordered by dense fringe of long bristle-like setae. Labium ventrally with short, flattened setae maximally 6.5–7.0× longer than wide, irregularly distributed along proximal margin of paraglossae (Fig. 1I). Dorsal side of first segment of labial palp with a group of more than 30 long pointed and short bluntly pointed setae; group of short setae not distinctly separated from long setae situated more proximally on first segment of labial palp (Fig. 1H).

*Thorax.* Yellowish-brown to light dirty olive; base of wing pads with diffuse whitish maculation; two unclear whitish spots on mesonotum laterally; indistinct elongated maculae centrally. Pleural and ventral side paler than dorsal. Legs pale, whitish-yellow to yellow or slightly olivaceous, without any distinct ornamentation. Coxae and trochanters of same colour as other leg segments. Surface of legs covered with elongated bristles, spatulate and hair-like setae and small setae with star-like distal end (up to 15 μm long). Forecoxae with at least 30 long bristle-like setae distally and at least 20 long stout setae on inner margin. Foretrochanters with tuft of dense, long bristle-like setae distally. Femora unicoloured, occasionally with small diffuse spot distally. Forefemora length 2.0–2.1× of their width; dorsal surface covered with few flattened setae of various length, arranged in irregular sparse rows near filtering setae centrally; sparse row of stout bristle-like setae extends along 1/3–1/4 of length of outer margin, and 13–17 short bluntly pointed setae in submarginal area (Fig. 2A). Filtering setae of femora and tibiae brown, unicoloured. Foretibiae with sparse setae along distal third of ventral side of foretibia (Fig. 2F); dorsal side with 3–4 setae arranged in irregular row in subapical part (Fig. 2G). Foretarsi with 2–3 bluntly pointed setae distally. Foreclaws moderately sclerotised at apex, with 4–5 denticles. Middle and hind coxae and trochanters with sparse setae of different size irregularly on surface. Femora of middle and hind legs with dense rows of long (longer than half of femur width) hair-like setae along 1/3–1/2 of posterior margin of femora; numerous thicker flattened and spine-like setae along posterior margin of proximal half of femora; small sparse spatulate setae scattered on dorsal surface of femora, forming irregular rows centrally; distal margin with setae of same shape, concentrated proximally and centrally, alternating with few elongated hair-like setae (Fig. 2B–E). Tibiae of middle and hind legs with few short hair-like and spatulate setae irregularly grouped subapically and apically. Tarsi of middle and hind legs with 4–5 elongated bluntly pointed or rounded setae apically, 6–7 hair-like setae along inner margin (as long as tarsi or shorter), alternating with 2–3 shorter stout setae subapically. Claws of middle and hind legs with 5–7 denticles.

*Abdomen* (Fig. 3). Yellowish-brown to dirty olive; indistinct diffuse pale maculae laterally; terga darker than sterna. Posterolateral processes relatively narrow, present on abdominal segments II–IX; processes on segment II indistinct. Apices of posterolateral processes well sclerotised. Posterolateral processes on segments IV–IX prominent, with outer margins nearly straight, slightly diverging from body axis; posterolateral processes of segment IX largest, with axes nearly parallel to body axis (Fig. 3G). Terga II–IX with unpaired posteromedial protuberance, rounded apically (Fig. 3A, B). Surface of terga covered evenly by very short spatulate setae rounded apically (Fig. 3F); number of setae on surface of terga gradually decreases from tergum II to IV; few flattened spatulate setae rounded apically (8–12 μm long; 9–10 μm wide) cover also posterolateral processes and terga I, V–X. Sterna covered with posteromedial groups of flattened setae, rounded apically (Fig. 3D, E). Length of these setae varies from 15–75 μm on sterna II and III; up to 100 μm on sterna IV–VIII; size of flattened setae gradually increases from sterna II to VII; individual setae are up to 10–15× longer than wide. Large group of setae assembled predominantly posteromedially on sterna II–VIII. Sternum IX covered only with sparse spatulate setae (up to 12 μm long), few elongated flattened setae up to 20 μm long, and numerous hair-like setae. Sternum IX with deep U-shaped incision posteriorly between two pointed, strongly sclerotised processes.

*Gills* (Fig. 4). Gill I significantly larger than following gills, rounded and symmetric, without ridge near outer margin; gills II–VII circular. Size ratios between gills I and IV approximately 1 : 0.7 (length) and 1 : 0.6 (width). All gill pairs equipped with bundle of whitish filaments, usually shorter than respective gill plate. Dorsal surface of gill I with sparse flattened setae concentrated mainly proximally (Fig. 4C). On lateral margins of gill plates, setae relatively short; setae bordering inner part of distal margin slightly longer (up to 25–30 μm long). Ventral surface of gill I with few isolated submarginal setae distally (up to 25 μm long, Fig. 4D); these setae strongly plumose, similar to setae occurring on ventral surface of following gill pairs. Sparse setation on dorsal and ventral surfaces of gills II–VII; dorsal surface covered with short spatulate setae (Fig. 4A, E, G); ventral surface with relatively short plumose setae sparsely scattered centrally along outer margin of ventral cavity (Fig. 4B, F, H).

*Cerci* whitish, unicoloured, with inner marginal fringe of fine, hair-like setae. Paracercus vestigial, 5-segmented (Fig. 3H).

***Egg, imago, and subimago.*** Unknown.

#### Affinities.

Among the Palaearctic genera of Oligoneuriidae, attribution of *O. tuberculata* sp. nov. to the genus *Oligoneuriella* is obvious, based on the shape of head, legs, and gills (see Bauernfeind and Soldán 2012). In the worldwide key to Oligoneuriidae genera published by Edmunds (1961), *O. tuberculata* sp. nov. erroneously would key out to the Nearctic and Neotropical genus *Lachlania* Hagen, 1868 due to the presence of a highly reduced paracercus. Edmunds (1961) at that time had not been aware of any *Oligoneuriella* species with reduced paracercus, since such species were only described later (Al-Zubaidi et al. 1987, Sroka et al. 2015). Despite the superficial resemblance of *O. tuberculata* sp. nov. to *Lachlania*, they differ in their setation on the anterior head margin. In *O. tuberculata* sp. nov., there are long, bristle-like setae (as in all *Oligoneuriella*). In *Lachlania* these setae are short and spatulate, which represents a crucial larval apomorphy of *Lachlania* (see Kluge 2004). In fact, *Lachlania* phylogenetically represents a quite distant lineage from *Oligoneuriella* and *Oligoneuriopsis* (Massariol et al. 2017). Relatively long setae medially on sterna II–V in *O. tuberculata* sp. nov. suggest a closer relationship with *Oligoneuriopsis*, but a large lamella of gill I and a short row of setae on the posterior margin of femora excludes this attribution (see the discussion on *O. villosus* sp. nov. below).

Within *Oligoneuriella*, the most closely related species to *O. tuberculata* sp. nov. are *O. bicaudata*Al-Zubaidi et al. 1987 from Iraq and *O. pectinata* Bojková & Soldán, 2015 from Turkey. Both species share with *O. tuberculata* sp. nov. the reduction of the paracercus to only a few segments, large lamella of gill I compared to other gill pairs, and the extent of setation on the posterior margin of middle and hind femora forming long and dense fringe. Nevertheless, *O. tuberculata* sp. nov. can be differentiated from both species by several characters:

**(i)** The first gill plate is markedly larger than gill pairs II–VII, nearly circular and symmetric, without a ridge near the outer margin in *O. tuberculata* sp. nov. In contrast, *O. pectinata* has an oval gill I, only slightly larger than the remaining gill pairs, with an indistinct ridge close to the outer margin (see Fig. 4C, D; Sroka et al. 2015: 341, fig. 46a, b). In *O. bicaudata*, the size ratio of gill I and remaining gill pairs is similar to *O. tuberculata* sp. nov., although the shape of gill plates is slightly different. In *O. tuberculata* sp. nov., the gill plate I is more circular (Fig. 4C, D), whereas in *O. bicaudata* it is rather elongated, narrowing proximally (Al-Zubaidi et al. 1987: fig. 5).

**(ii)** The setation on the surface of gills: in *O. tuberculata* sp. nov., dorsal and ventral surface of the gill I is equipped with very few flattened setae (Fig. 4C, D), whereas in *O. pectinata* relatively dense setation occurs, especially on the ventral surface (Sroka et al. 2015: 341, fig. 46a, b). The setae on the ventral surface of gills are strongly plumose in both species. However, these setae are shorter (up to 25 μm long) in *O. tuberculata* sp. nov. than in *O. pectinata* (up to 32 μm long) (Sroka et al. 2015: 344, fig. 66a). Lateral margins of the first gill are equipped with rich, long setae in *O. pectinata*, in contrast to *O. tuberculata* sp. nov. with relatively short setae along margins (Fig. 4C, D). Details of gill setation are unknown for *O. bicaudata*.

**(iii)** All three species can be separated by the shape of abdominal segments. Unpaired posteromedial protuberances on terga are characteristic for *O. tuberculata* sp. nov. (Fig. 3A, B), as they are lacking in *O. pectinata* and Al Zubaidi et al. (1987) did not mention any protuberances on the terga of *O. bicaudata*. Thus, given the conspicuousness of this character, we consider it plausible to assume that the protuberances are absent in this species. Moreover, the protuberances are missing in the material identified as *O. bicaudata* collected near the type locality of this species in Iraq (Al-Saffar, pers. comm). Furthermore, posterolateral processes of the abdominal segments II–VII are relatively narrow, slightly bent outwards in *O. tuberculata* sp. nov. (Fig. 3G), in contrast to relatively robust and straight processes in *O. pectinata*. In *O. bicaudata*, these processes are very thin, curved inward apically, especially on segment IX (Al-Zubaidi et al. 1987, fig. 2).

**(iv)** All three species have a pale body colouration and an inconspicuous colour pattern on the abdominal terga. However, *O. pectinata* is slightly darker, with a pair of diffuse median spots on terga, whereas *O. tuberculata* sp. nov. is characterised by the presence of diffuse maculae laterally. Abdomen of *O. bicaudata* is pale brown, without any distinct pattern (Al-Zubaidi et al. 1987).

An analysis of diagnostic characters based on adults is impossible at present, as adults are not known for *O. tuberculata* sp. nov. and *O. bicaudata*. The colouration pattern and size of eyes would slightly differ in fully mature larvae from the material described herein.

### Oligoneuriopsis villosus

Taxon classificationAnimaliaEphemeropteraOligoneuriidae

Bojková, Godunko, & Staniczek sp. nov.

C9A78F598F8A5837AC8174AC0A9040E3

http://zoobank.org/3980D827-DA07-40CE-BE30-F997FE5EDBCC

**Figure 5.**
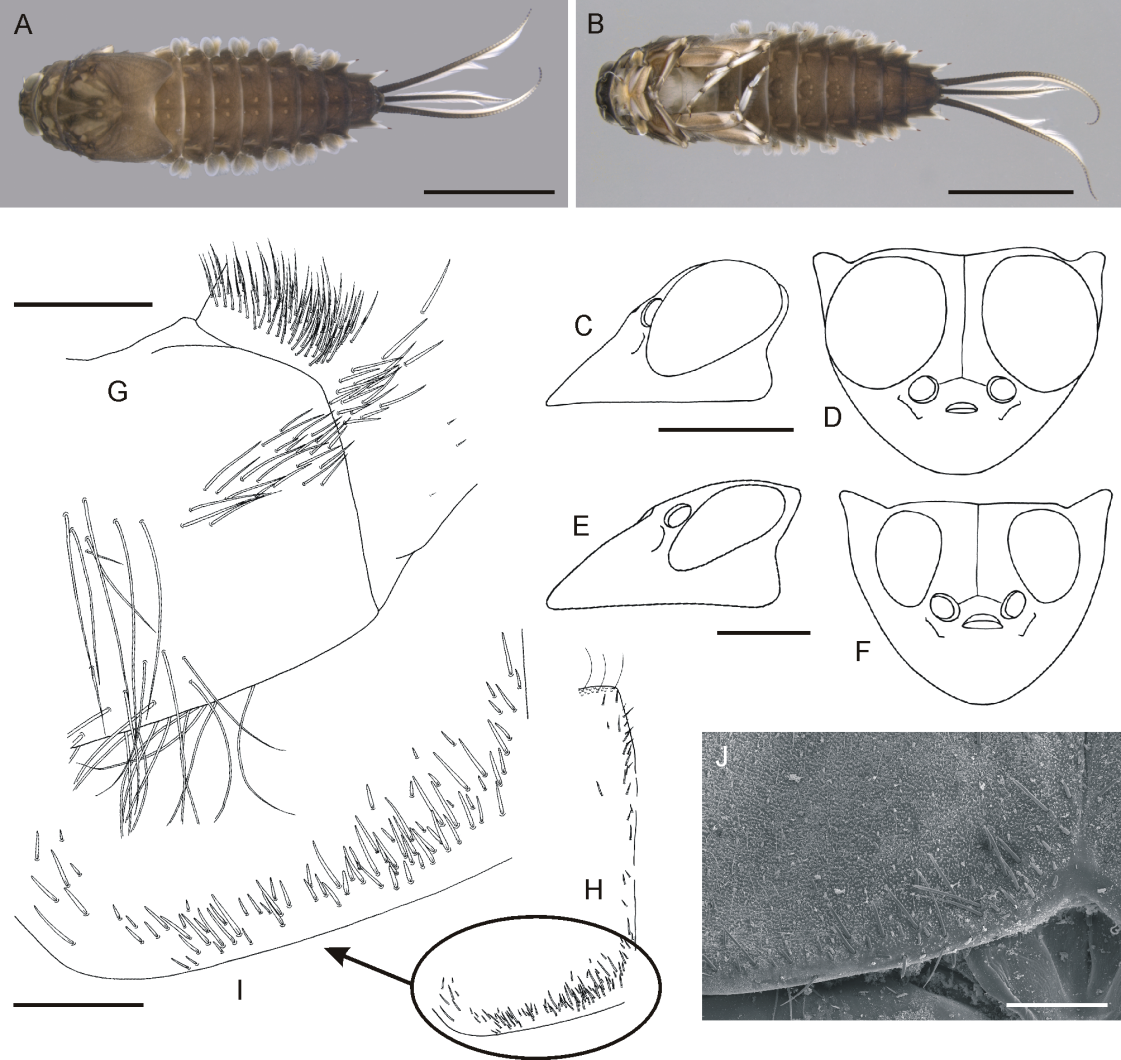
*Oligoneuriopsis villosus* sp. nov., larvae **A** habitus in dorsal view **B** habitus in ventral view **C** head of male in lateral view **D** head of male in dorsal view **E** head of female in lateral view **F** head of female in dorsal view **G** detail of setae on distal part of labial palp segment I in dorsal view **H–I** setae on paraglossae in ventral view **J** detail of basal portion of paraglossae under SEM in ventral view. Scale bars: 5 mm (**A, B**); 1 mm (**C–F**); 0.2 mm (**G–J**). Same scale bar for **C** and **D**. Same scale bar for **E** and **F**.

**Figure 6.**
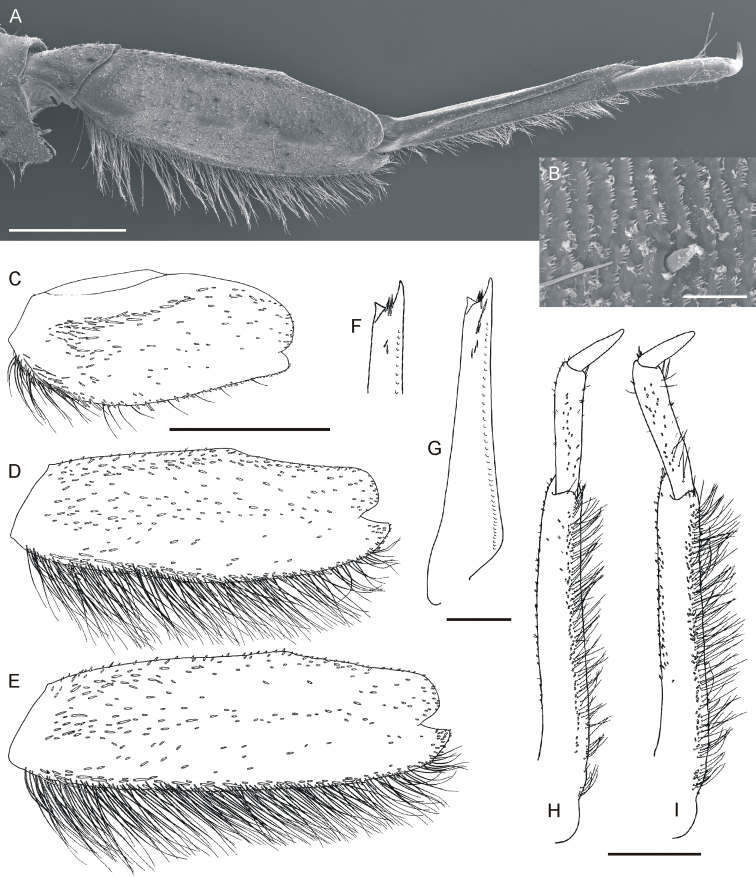
*Oligoneuriopsis villosus* sp. nov., larvae, legs **A** hind leg under SEM in dorsal view **B** micro sculpture of leg cuticula under SEM in dorsal view **C** forefemur in dorsal view **D** middle femur in dorsal view **E** hind femur in dorsal view **F** apical part of foretibia with subapical setae in ventral view **G** shape of foretibia with subapical setae in dorsal view **H** middle tibia and tarsus in dorsal view **I** hind tibia and tarsus in dorsal view. Scale bars: 1 mm (**A, C–E**); 0.02 mm (**B**); 0.5 mm (**F–I**). Same scale bar for **C, D** and **E**. Same scale bar for **F** and **G**. Same scale bar for **H** and **I**.

**Figure 7.**
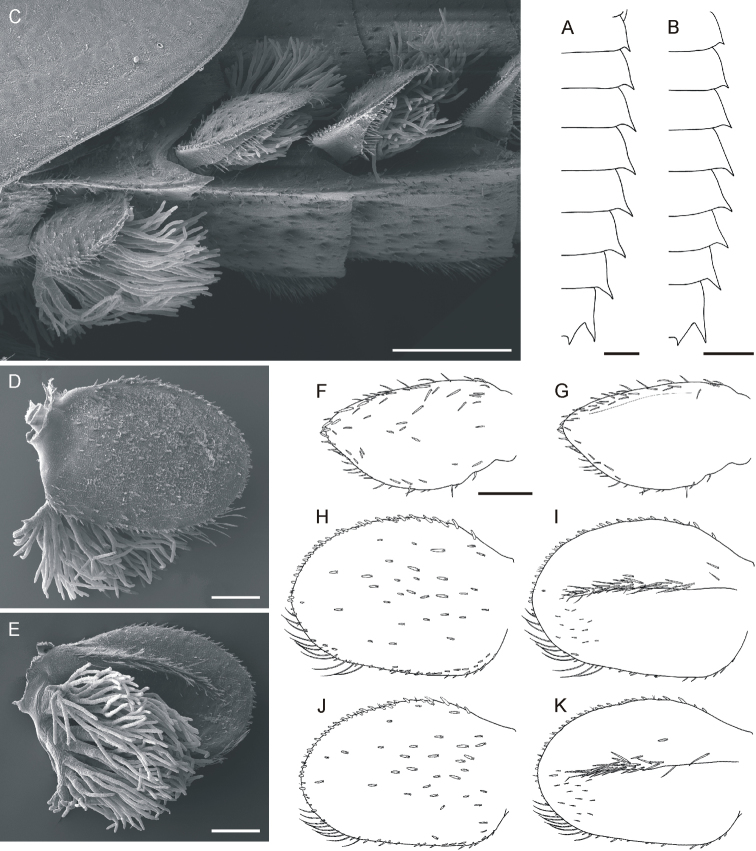
*Oligoneuriopsis villosus* sp. nov., larvae, abdomen and gills **A** abdomen outline, male **B** abdomen outline, female **C** proximal portion of abdomen under SEM in lateral view **D** gill IV under SEM in dorsal view **E** gill IV under SEM in ventral view **F** gill plate I in dorsal view **G** gill plate I in ventral view **H** gill plate IV in dorsal view **I** gill plate IV in ventral view **J** gill plate VII in dorsal view **K** gill plate VII in ventral view. Scale bars: 1 mm (**A, B**); 0.5 mm (**C**); 0.2 mm (**D–K**). Same scale bar for **F–K**.

**Figure 8.**
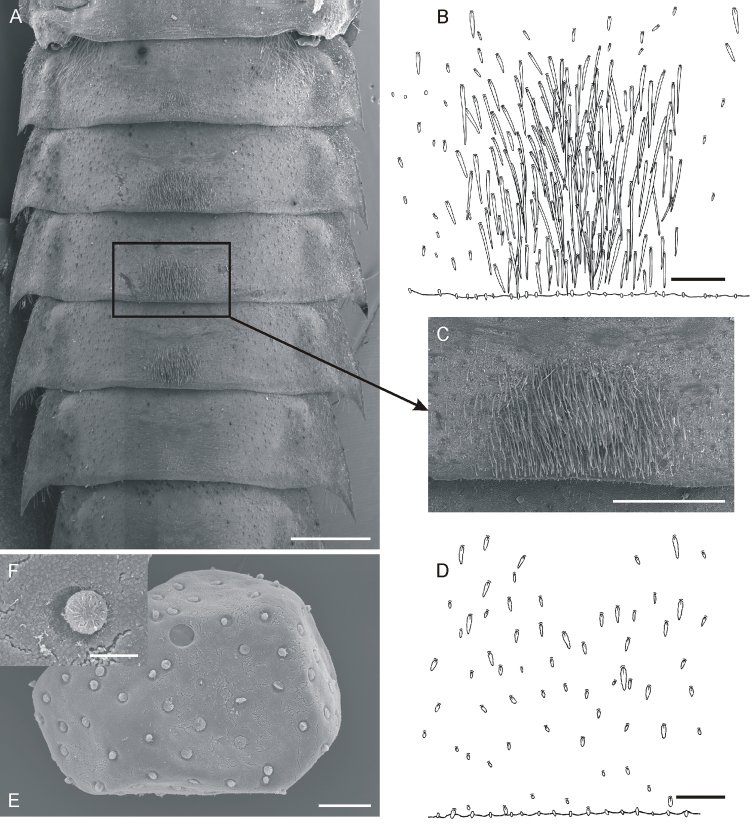
*Oligoneuriopsis villosus* sp. nov., larvae and eggs **A** general view of basal part of abdomen under SEM in ventral view **B** detail of setae on sternum IV **C** detail of setae on sternum IV under SEM**D** detail of setae on surface of terga **E** egg, general view **F** detail of egg chorionic structure. Scale bars: 1 mm (**A**); 0.1 mm (**B, D**); 0.5 mm (**C**); 0.05 mm (**E**); 0.01 mm (**F**).

Figures 5 , 6 , 7 , 8 , Table 2

#### Etymology.

The name of the new species originates from Latin, meaning hairy, and refers to the dense setation along outer margins of femora and tibiae.

#### Type material.

***Holotype***: Male larva, IRAN, Khuzestan Province, right tributary of Marun Rud River, W of Bagh Malek, 31°31.23'N, 49°49.31'E (locality no. 93), 585 m a.s.l., 29.04.2017, leg. Bojková J., Soldán T. & Imanpour Namin J. The holotype is deposited in IECA.

***Paratypes***: 64 larvae, same locality as holotype, deposited in IECA. Eight larvae, IRAN, Khuzestan Province, Balarud River (right tributary of Dez River), N of Andimeshk, 32°35.29'N, 48°17.32'E (locality no. 80), 230 m a.s.l., 26.04.2017, leg. Bojková J., Soldán T., & Imanpour Namin J., deposited in IECA.

70 larvae, IRAN, Hormozgan Province, Shamil River, Shamil, 27°29.66'N, 56°52.25'E, 63 m a.s.l., 30.04.2017, leg. Staniczek A.H., Godunko R.J., Pallmann M., & Nejat F. (20 larvae deposited in IECA, 20 larvae deposited in SNHM, 20 larvae deposited in SMNS under accessory numbers SMNS_EPH_7555_B_1 and SMNS_EPH_7555_B_2, including DNA voucher specimens with inventory numbers specified in Table 1, and 10 larvae deposited in MMTT_DOE).

110 larvae, IRAN, Hormozgan Province, Roudan River, 5 km N of Dehbarez, 27°28.46'N, 57°15.28'E, 217 m a.s.l., 30.04.2017, leg. Staniczek A.H., Godunko R.J., Pallmann M., & Nejat F. (20 larvae deposited in IECA, 70 larvae deposited in SNHM, and 20 larvae deposited in SMNS under inventory numbers SMNS_EPH_7550_B_1 and SMNS_EPH_7550_V_1–5, including DNA voucher specimens with inventory numbers specified in Table 1).

#### Localities and biology.

**Figure 9.**
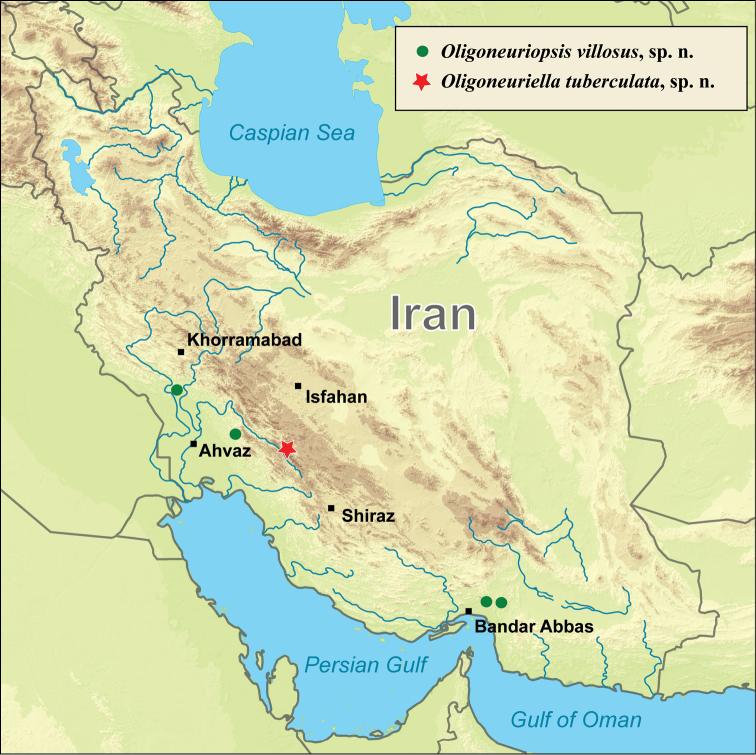
Map of Iran with localities of *Oligoneuriella tuberculata* sp. nov. (star) and *Oligoneuriopsis villosus* sp. nov. (circles).

Larvae were collected in two rivers at the southern slopes of Zagros Mountains (right tributary of Marun Rud River and Balarud River) and two other localities (Shamil River and Roudan River) in Hormozgan Province at the western edge of Makran, a semi-desert coastal strip, which stretches along the Gulf of Oman (Fig. 9). Extensive land use is present in most parts of the drainage area, mainly for agricultural and residential matters in all localities. In Marun Rud River basin, the agricultural use is more intense than in other localities and there are several urban settlements in the drainage area of this river.

**Figure 10.**
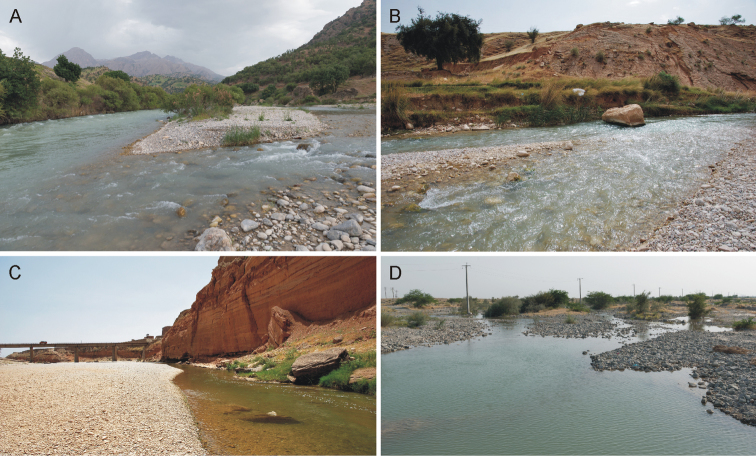
Photos of the localities with occurrence of *Oligoneuriella tuberculata* sp. nov. and *Oligoneuriopsis villosus* sp. nov. **A** Marbor River near Kata (type locality of *O. tuberculata* sp. nov.) **B** right tributary of Marun Rud River near Bagh Malek (type locality of *O. villosus* sp. nov.) **C** Balarud River near Andimeshk (locality of *O. villosus* sp. nov.) **D** Shamil River near Shamil (locality of *O. villosus* sp. nov.).

The species was generally found in shallow river sections (up to 50 cm) with a riverbed composed of coarse and fine gravel, sometimes in combination with cobbles. The localities with the occurrence of this species represented rather small rivers; however, the stream width varied from 4 to 40 m, depending on the river discharge rates at the end of March (Fig. 10B–D).

Larvae were predominantly distributed in river sections with accelerated current and with a minimum amount of alluvial silt. Large larvae (more than 1 cm) were found along stony margins of riffles, while small and medium-sized larvae (up to 1 cm) dwelt anywhere in the riffles.

Water quality at collection sites in Khuzestan Province was good, with pH 8.0–8.4, conductivity 620–760 µS.cm^-1^, salinity 0.3–0.4 ‰, and temperature 21–29 °C (conductivity and salinity measured on two localities only – the tributary of Marun Rud River and Balarud River). At collection sites in Hormozgan Province, abiotic factors were as following: conductivity 1520-1646 µS.cm, salinity 0.8 ‰, and temperature 32–34 °C.

Emergence of the species can be expected between June and July, as most of larvae occurring in all the localities were small and/or middle-sized at the time of collection. Mayfly taxa found in the same localities included: *Baetis* s. str. (Baetidae), *Labiobaetis* sp. (Baetidae), *Nigrobaetis* sp. (Baetidae), *Rhithrogena* sp. (Heptageniidae), *Electrogena* sp. (Heptageniidae), *Choroterpes* sp. (Leptophlebiidae), *Caenis* sp. (Caenidae), and *Prosopistoma* sp. (Prosopistomatidae).

#### Diagnosis.

According to the combination of following diagnostic characters, *O. villosus* sp. nov. can be distinguished from all other representatives of the genus *Oligoneuriopsis* worldwide:

• head widest across posterolateral corners (Fig. 5D, F);

• row of long setae along all length of middle- and hind femora and tibiae (Fig. 6A, D, E, H, I);

• posteromedian projections on abdominal terga absent (Fig. 5A);

• colouration of abdominal terga dark brown with two pale dots medially (Fig. 5A);

• setae on surface of terga and gills elongated and bluntly pointed (Figs 7F, H, J, 8D);

• posterolateral projections of abdominal segments diverging from body axis (Fig. 7A, B);

• posteromedial setae on sterna III–IV very long and dense, some more than 20× longer than wide (Fig. 8A–C);

• first gill plate markedly smaller than the remaining pairs (Fig. 7F, G);

• setae on inner distal margin of gill plates II–VII long (Fig. 7H–K);

• setae on ventral surface of gill plates near inner distal margin present (Fig. 7I, K);

• paracercus fully developed (Fig. 5A, B);

• caudal filaments dark in proximal half, distinct dark band in the middle missing (Fig. 5A, B).

#### Description.

***Larva.*** Mature larvae: body length 13–16 mm (female), 11–12 mm (male), length of cerci approximately 0.4–0.5× body length, paracercus slightly shorter.

*Colouration* (Fig. 5A, B). Head, thorax and abdomen greyish dark brown with distinctive light (yellowish) ornamentation. When fixed in ethanol, general colouration paler, brown. Legs yellowish with distinctive brown ornamentation forming bands. This colouration apparent only in larger larvae, more than 1 cm long. Smaller larvae uniformly pale, yellowish.

*Head*. Greyish dark brown, foremargin of head darker, occipital area with yellowish ornamentation forming four longitudinal stripes merging behind ocellar part of head. Antennae yellowish brown proximally, brown distally. Head width/length 1 : 1.2 (Fig. 5C–F). Eyes black, slightly exceeding contour of head in dorsal view in males; distance between eyes 2.2× narrower than eye width in males and 1.5× wider than eye width in females (Fig. 5D, F). Foremargin of head dark, bordered by dense fringe of long bristle-like setae. Labium ventrally with numerous flattened setae of different length not arranged in rows along proximal margin of paraglossae (Fig. 5H–J). Similar setae also situated sparsely along inner margin of paraglossae (Fig. 5H). Dorsal side of first segment of labial palp with group of 15–25 thin setae distally (Fig. 5G).

*Thorax*. Greyish dark brown, with yellowish spots submedially on prothorax and yellowish spots and longitudinal stripes on mesothorax. Pleural and ventral part of thorax brown. Legs yellowish-brown, with distinct brown ornamentation (Fig. 5B). Coxae and trochanters brown. Proximal half of femora brown, distal half yellowish-brown. Tibiae with narrow brown bands distally and proximally, tarsi with narrow brown band distally. Forecoxae distally with numerous long, bristle-like setae and with about 15 long, thin hair-like setae on inner margin. Foretrochanters distally with numerous long, bristle-like setae. Forefemora length 2–2.5× width; dorsal surface of forefemora covered with scattered flattened setae of various length; dense group of pointed long flattened setae submarginally near filtering setae; outer margin of forefemora bordered by conspicuous fringe of long hair-like setae (Fig. 6C). Filtering setae of femora and tibiae brown. Foretibiae dorsally with irregular row of bristle-like setae in distal quarter (Fig. 6G); ventrally with 2–3 subapical setae and several setae apically (Fig. 6F). Foretarsi distally with groups of pointed setae. Foreclaws heavily sclerotised at apex, with 6–7 denticles. Middle and hind femora and tibiae with dense fringe of long, hair-like setae along entire length of outer margin (Fig. 6A, D, E, H, I). Some hair-like setae present on outer margin of tarsi as well (Fig. 6I). Fringes of hair-like setae on femora and middle and hind tibiae are apparent also in (pale) early-instar larvae.

*Abdomen*. Greyish dark brown, with pair of submedian smudged yellowish spots and conspicuous sublateral yellowish pattern (Fig. 5A). Ventral part of abdomen greyish dark brown, with conspicuous yellowish pattern (Fig. 5B). Posterolateral processes present on abdominal segments II–IX; bent outwards, apices slightly inwards (Fig. 7A, B). Terga sparsely covered with flattened setae of various sizes mixed with numerous microtrichia, largest setae elongated and bluntly pointed apically (Fig. 8D). Some sterna with densely assembled setae posteromedially (most prominent on sterna III–IV, present also on sternum V). Individual setae distinctly elongated, some more than 20× longer than wide, see Fig. 8A–C). Sternum IX with wide rounded incision posteriorly, equipped with tiny spines.

*Gills* (Fig. 7). Gill I asymmetric, bluntly pointed apically (Fig. 7F, G), markedly smaller and more elongated than following gills, which are oval and almost symmetric (Fig. 7D, E, H–K). Size ratio between gill I and IV approximately 0.8 : 1 (length) and 0.7 : 1 (width). All gill pairs equipped with bundle of whitish filaments slightly shorter than respective gill plate (filaments are slightly longer in gill I). Dorsal surface of gill I covered with elongated setae of various size (Fig. 7F). Ventral surface of gill I with submarginal setae only (Fig. 7G). Dorsal surface of gills II–VII with sparsely scattered, elongated, and bluntly pointed setae (Fig. 7D, H, J). Similar setae occurring also on margins of gill plates. Setae bordering inner part of distal margin relatively long (Fig. 7H–K). Ventral surface of gill plates II–VII with numerous hair-like setae along outer margin of ventral cavity (Fig. 7E, I, K). These setae slightly plumose. Second group of minute setae situated near inner distal margin (Fig. 7I, K).

*Cerci* dark brown, lighter towards apex, with inner marginal fringe of fine hair-like setae. Paracercus reaching approx. 2/3 of cerci length, with dense setation laterally (Fig. 5A, B).

***Egg.*** Shape of eggs studied deformed due to extraction from mature larvae (Fig. 8E). Eggs 250–320 μm long and 190–240 μm wide; chorionic surface covered with circled terminal fibre clusters (diameter 10 μm). Micropyle shallow, circular; sperm guide well apparent (Fig. 8E). Terminal fibre clusters finely sculptured, with leaf-like, flat microstructures (Fig. 8F).

***Imago and subimago.*** Unknown.

#### Affinities.

The establishment of the generic attribution of *O. villosus* sp. nov. is not straightforward, although affinities to the genera *Oligoneuriella* Ulmer, 1924 and *Oligoneuriopsis* Crass, 1947 are obvious based on the shape of head, legs, and gills (see Edmunds 1961; Bauernfeind and Soldán 2012). However, differences between *Oligoneuriella* and *Oligoneuriopsis* are very subtle and the separation of these genera is not clear. The first description of the genus *Oligoneuriopsis* was published by Crass (1947), with the type species *Oligoneuriopsis lawrencei* Crass, 1947, mainly based on the adult stage. Its larva was not described in detail, but briefly commented as being identical to larvae described and depicted earlier by Barnard (1940), who assigned them to “*Elassoneuria trimeniana*”. This opinion is followed by later authors, for example Demoulin (1970). Edmunds (1961) considered *Oligoneuriopsis* as closely related to *Oligoneuriella*. In the larval key to genera, the presence of the lamella of gill I in *Oligoneuriella* and its absence in *Oligoneuriopsis* was used as the crucial diagnostic character separating the genera (Edmunds 1961). Nevertheless, Agnew (1980) clearly stated that this character is not valid, since a small lamella is present also in *Oligoneuriopsis*, including the type species *O. lawrencei*. Instead, he proposed the arrangement of the setae medially on abdominal sterna being more developed in *Oligoneuriopsis* than in *Oligoneuriella* as a reliable diagnostic character. Bauernfeind and Soldán (2012) mentioned more conspicuous, very long setae medially on sterna, lamella of gill I reduced, membranous, and cerci with dark band in the middle as diagnostic for *Oligoneuriopsis*. In our view, the length of posteromedial setae on sterna probably mostly represents a reliable character, although it varies, and the setae are reported 3–6× longer than wide in known species of *Oligoneuriella* (Sroka et al. 2015) and even more than 10× longer than wide in *Oligoneuriella tuberculata* sp. nov., described above. However, in *O. villosus* sp. nov., some of these setae are more than 20× longer than wide (Fig. 8B, C), which fulfils the criteria for *Oligoneuriopsis* and roughly also agrees with our observation of setae length in *Oligoneuriopsis skhounate* from North Africa. We observed that the lamella of gill I in *O. skhounate* is small-sized compared to other gills, but structurally not different. The same condition applies also to *O. villosus* sp. nov. (Fig. 7C). A clearer morphological definition of *Oligoneuriopsis* would be possible after a more thorough study of the Afrotropical material, including the type species *O. lawrencei*, as already pointed out by Bauernfeind and Soldán (2012). Ideally such a study should also include adults, since several adult characters separating *Oligoneuriella* from *Oligoneuriopsis* have been published, mostly concerning wing venation and male genitalia (Bauernfeind and Soldán 2012).

At this time, we consider it justified to assign the new species to *Oligoneuriopsis* based on following characters shared with *O. skhounate* (and presumably also with other Afrotropical representatives of *Oligoneuriopsis*): (i) setae posteromedially on abdominal sterna very long, some more than 20× longer than wide; (ii) lamella of gill I significantly smaller than remaining gill pairs; and (iii) row of setae along entire length of posterior margin of femora and outer margin of tibiae on middle and hind legs. The second and third characters are also depicted in the figures provided by Barnard (1940), presumably representing the larva of *O. lawrencei*, which supports their diagnostic value for the separation of *Oligoneuriopsis* from *Oligoneuriella*.

In the genus *Oligoneuriopsis*, six species have been described up to now (Bauernfeind and Soldán 2012). Five of them are distributed exclusively in the Afrotropics; only a single species, *O. skhounate*, occurs also in the Palaearctic (North Africa and the Iberian Peninsula). The occurrence of Afrotropical species in Iran is extremely unlikely (even more when taking into account that most species are known from South Africa only). Nevertheless, all these species can be morphologically distinguished from *O. villosus* sp. nov. (except *O. dobbsi* (Eaton, 1912) with unknown larva).

*Oligoneuriopsis lawrencei* Crass, 1947 differs in the shape of head, widest anterior to the eyes (Agnew 1973, fig. 1) contrary to *O. villosus* sp. nov. with the head widest across posterolateral corners (Fig. 5D, F). Furthermore, *O. lawrencei* possesses very long setae on margin of gill plates (Agnew 1980, fig. 8). *O. jessicae* Agnew, 1973 profoundly differs by having posteromedial spine-like projections on abdominal terga (Agnew 1973, fig. 1), missing in *O. villosus* sp. nov. (Fig. 5A). *O. elisabethae* Agnew, 1973 differs from *O. villosus* sp. nov. in colouration. *O. elisabethae* exhibits a uniform light brown colour, without any indication of an abdominal pattern (Agnew 1973), whereas *O. villosus* sp. nov. possesses a distinct pair of submedian, smudged, yellowish spots and a conspicuous, sublateral, yellowish pattern on the dorsal side of abdomen (Fig. 5A).

The nearest distributed congeneric species *O. skhounate* is morphologically similar to *O. villosus* sp. nov. It even shares the colouration pattern of legs with alternating darker and lighter bands (Fig. 5B; Dakki and Giudicelli 1980, figs 28, 29). Nevertheless, several morphological characters allow the separation of these two species: (i) setae on the surface of gills and terga are shorter in *O. skhounate*, longer and more pointed in *O. villosus* sp. nov. (Fig. 8D); (ii) colouration is different, prominent posteromedian pale spot on terga in *O. skhounate* (Dakki and Giudicelli 1980, fig. 20) is missing in *O. villosus* sp. nov. (Fig. 5A). Moreover, the dark band at half length of caudal filaments, depicted for *O. skhounate* (Dakki and Giudicelli 1980; fig. 20), is also missing in *O. villosus* sp. nov. (Fig. 5A, B); (iii) in *O. skhounate*, posterolateral projections of abdominal segments are oriented parallel to the body axis, and lateral margins of individual segments are convex (Dakki and Giudicelli 1980, fig. 20). In *O. villosus* sp. nov., posterolateral projections of abdominal segments are diverging from the body axis, with lateral margins straight or slightly concave (Fig. 7A, B).

**Figure 11.**
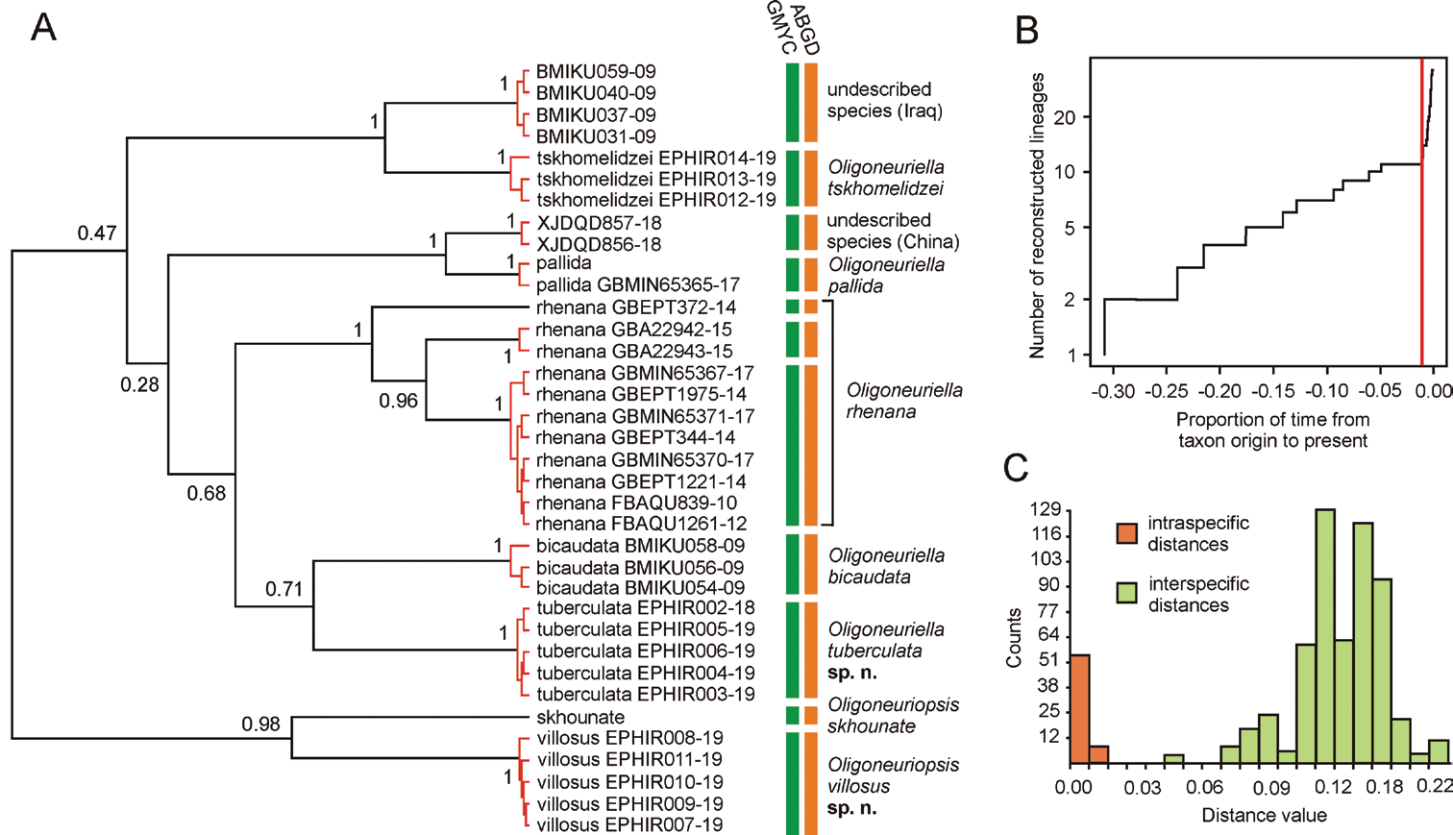
Results of the molecular species delimitation: **A** maximum clade credibility COI gene tree. The red branches represent species delimited by GMYC. Columns on the right illustrate groups delimited by both approaches used **B** lineage through time plot, generated by GMYC. The red vertical line indicates the threshold time between inter- and intraspecific branching **C** distribution of distances calculated by ABGD.

Al-Zubaidi and Al-Kayatt (1986) without further details already reported findings of *Oligoneuriopsis* sp. from north Iraq. An unidentified species of *Oligoneuriopsis* was also collected recently from the same area and deposited in the Bold database as *Oligoneuriopsis* sp. MAA01. This record contains two barcode sequences, which are not publicly accessible, but exhibiting 90.03–90.63% similarity to the haplotypes of *O. villosus* sp. nov. sequenced in the present study. This level of similarity rather corresponds with interspecific distances found in Oligoneuriidae (see Fig. 11C). Therefore, we may assume the existence of more *Oligoneuriopsis* species in the Middle East.

#### Molecular species delimitation.

The ABGD analysis of the COI distance matrix generated 11 stable groups (Fig. 11A). The mean genetic distances within groups generated by ABGD ranged between 0.003 and 0.011 and the mean distances between groups between 0.060 and 0.234; for the histogram of distances see Fig. 11C.

The GMYC model recognised 11 ML entities, with confidence interval 11–11 (Fig. 11A, B). We used single threshold GMYC, since multiple threshold is prone to over splitting species that are not evenly sampled throughout its distributional area (Fujisawa and Barraclough 2013; Hrivniak et al. 2019). The Maximum Likelihood of GMYC model was 197.4186, compared to the likelihood of the null model 181.5407. The likelihood ratio test significantly rejected the null model expecting uniform coalescent branching rates across entire tree (likelihood ratio=31.75579, p=1.271505e-07).

The clusters delimited as distinct species were congruent in both approaches. Both methods of the molecular species delimitation also unambiguously supported the designation of *O. tuberculata* sp. nov. and *O. villosus* sp. nov., which were recovered as distinct units in both analytical approaches. Apart from the two new species described herein, previously known species recognised as distinct entities in both ABGD and GMYC included *O. pallida*, *O. bicaudata*, *O. tskhomelidzei*, and *O. skhounate*. The species identified morphologically as *O. rhenana* was split into three putative COI species, which indicates that this widely distributed taxon (occurring in most of Europe, see Bauernfeind and Soldán 2012) actually represents a species complex. The putative cryptic species within *O. rhenana* were left undescribed for the moment, since extensive material was not at our disposal. Within the dataset, a further two unidentified putative species were recognised, one from China and one from Iraq. These clusters possibly represented a further two undescribed species, left unnamed for the time being.

The analysis of COI corroborated the affinity of *O. villosus* sp. nov. to *Oligoneuriopsis*. From the taxa included, *O. villosus* sp. nov. exhibited the highest sequence similarity with *O. skhounate* from Southern Spain. However, the distance between *O. villosus* sp. nov. and *O. skhounate* clearly separated them into two distinct species. Regarding the affinities of *O. tuberculata* sp. nov., *O. bicaudata* was recovered as the most closely related species from the ones sequenced, which is in accordance with the morphological character distributions.

## Supplementary Material

XML Treatment for Oligoneuriella tuberculata

XML Treatment for Oligoneuriopsis villosus
